# 3-Nitro­phenol–1,3,5-triazine-2,4,6-tri­amine (2/1)

**DOI:** 10.1107/S1600536813011148

**Published:** 2013-05-04

**Authors:** V. Sangeetha, N. Kanagathara, G. Chakkaravarthi, M.K. Marchewka, G. Anbalagan

**Affiliations:** a Department of Physics, D.G. Vaishnav College, Chennai 600 106, India; b Department of Physics, Vel Tech Multi Tech Dr Rangarajan Dr Sakunthala Engineering College, Chennai 600 062, India; c Department of Physics, CPCL Polytechnic College, Chennai 600 068, India; d Institute of Low Temperature and Structure Research, Polish Academy of Sciences, 50-950 Wrocław, 2, PO Box 937, Poland; e Department of Physics, Presidency College, Chennai 600 005, India

## Abstract

The asymmetric unit of the title compound, C_3_H_6_N_6_·2C_6_H_5_NO_3_, contains one melamine and two 3-nitro­phenol mol­ecules. The mean planes of the 3-nitro­phenol mol­ecules are almost orthogonal to the plane of melamine, making dihedral angles of 82.77 (4) and 88.36 (5)°. In the crystal, mol­ecules are linked *via* O—H⋯N, N—H⋯N and N—H⋯O hydrogen bonds, forming a three-dimensional network. The crystal also features weak C—H⋯π and π–π inter­actions [centroid–centroid distance = 3.9823 (9) Å].

## Related literature

For general background to melamine derivatives, see: Desiraju *et al.* (1990 ▶); Krische & Lehn (2000 ▶). For related structures, see: Kanagathara *et al.* (2012 ▶); Wang *et al.* (2007 ▶).
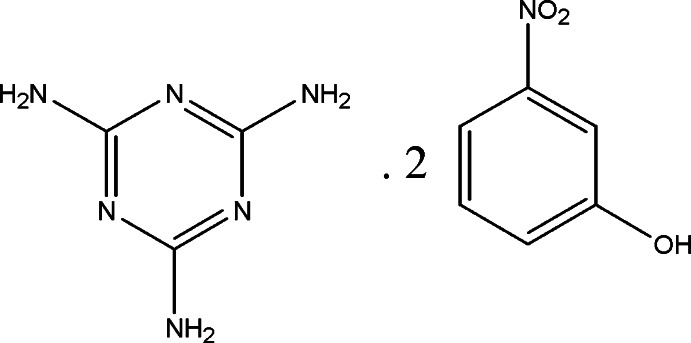


## Experimental

### 

#### Crystal data

C_3_H_6_N_6_·2C_6_H_5_NO_3_
*M* *_r_* = 404.36
Orthorhombic, 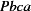

*a* = 15.5150 (6) Å
*b* = 12.9137 (6) Å
*c* = 17.8323 (6) Å
*V* = 3572.8 (2) Å^3^
*Z* = 8
Mo *K*α radiation
μ = 0.12 mm^−1^
*T* = 295 K
0.28 × 0.24 × 0.20 mm

#### Data collection

Bruker Kappa APEXII CCD diffractometer
Absorption correction: multi-scan (*SADABS*; Sheldrick, 1996 ▶) *T*_min_ = 0.967, *T*_max_ = 0.977
19568 measured reflections
4447 independent reflections
3352 reflections with *I* > 2σ(*I*)
*R*_int_ = 0.028

#### Refinement

*R*[*F* ^2^ > 2σ(*F* ^2^)] = 0.042
*wR*(*F* ^2^) = 0.119
*S* = 1.03
4447 reflections
295 parameters
8 restraints
H atoms treated by a mixture of independent and constrained refinement
Δρ_max_ = 0.24 e Å^−3^
Δρ_min_ = −0.20 e Å^−3^

### 

Data collection: *APEX2* (Bruker, 2003 ▶); cell refinement: *SAINT* (Bruker, 2003 ▶); data reduction: *SAINT*; program(s) used to solve structure: *SHELXS97* (Sheldrick, 2008 ▶); program(s) used to refine structure: *SHELXL97* (Sheldrick, 2008 ▶); molecular graphics: *PLATON* (Spek, 2009 ▶); software used to prepare material for publication: *SHELXL97*.

## Supplementary Material

Crystal structure: contains datablock(s) I, global. DOI: 10.1107/S1600536813011148/bh2476sup1.cif

Structure factors: contains datablock(s) I. DOI: 10.1107/S1600536813011148/bh2476Isup2.hkl

Supplementary material file. DOI: 10.1107/S1600536813011148/bh2476Isup3.cml

Additional supplementary materials:  crystallographic information; 3D view; checkCIF report

## Figures and Tables

**Table 1:** Hydrogen-bond geometry (Å, °) *Cg*3 is the centroid of the melamine tri­amine ring.

*D*—H⋯*A*	*D*—H	H⋯*A*	*D*⋯*A*	*D*—H⋯*A*
O1—H1⋯N3^i^	0.84 (1)	1.86 (1)	2.6907 (14)	176 (2)
O4—H4*A*⋯N2^ii^	0.83 (1)	1.87 (1)	2.6876 (14)	170 (2)
N5—H5*A*⋯N4^iii^	0.89 (1)	2.17 (1)	3.0594 (18)	178 (17)
N5—H5*B*⋯O1^iv^	0.87 (1)	2.25 (1)	2.9613 (16)	138 (15)
N7—H7*A*⋯O1^v^	0.88 (1)	2.32 (1)	3.1600 (17)	159 (14)
N7—H7*B*⋯O4^vi^	0.88 (1)	2.13 (1)	2.9180 (16)	149 (15)
C6—H6⋯*Cg*3^vii^	0.93	2.95	3.7504 (18)	145

Symmetry codes: (i) 
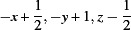
; (ii) 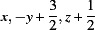; (iii) 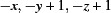; (iv) 
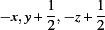
; (v) 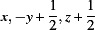; (vi) 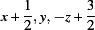; (vii) 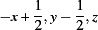.

**Fig. 1.**
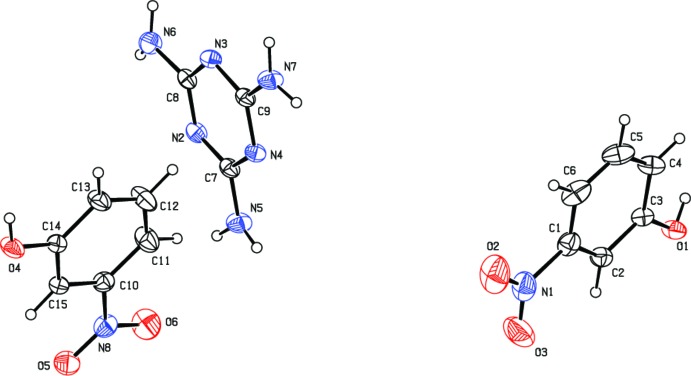
The molecular structure of the title compound, with 30% probability displacement ellipsoids for non-H atoms.

**Fig. 2.**
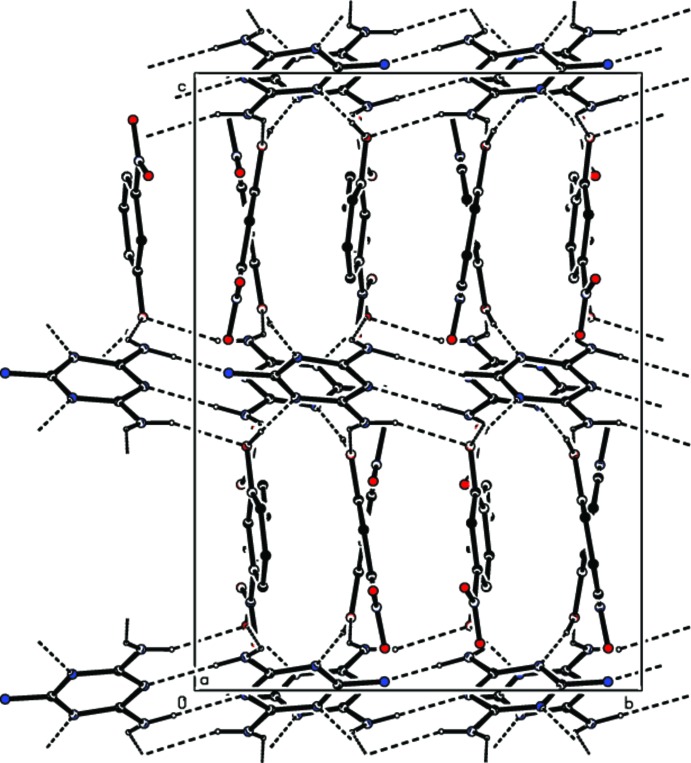
The packing of the title compound viewed down the a axis. Hydrogen bonds are shown as dashed lines.

## supplementary crystallographic information

### Comment

Melamine (1,3,5-triazine-2,4,6-triamine) and its derivatives can develop well defined non-covalent supramolecular nanoarchitectures *via* multiple hydrogen bonds by self-assembly of components containing complementary arrays of hydrogen-bonding sites (Desiraju, 1990; Krische & Lehn, 2000). The geometric parameters of the title compound (Fig. 1) are comparable to those reported for similar structures (Kanagathara *et al.*, 2012; Wang *et al.*, 2007). The mean planes of the two nitrophenol molecules (C1···C6) and (C10···C15) are almost orthogonal to the melamine (N2/C7/N4/C9/N3/C8) molecule, with dihedral angles of 82.77 (4) and 88.36 (5)°, respectively.

The crystal packing of the title compound is influenced by intermolecular O—H···N, N—H···N and N—H···O hydrogen bonds, as well as weak C—H···π (Table 1 and Fig. 2) and π–π interactions: *Cg*1···*Cg*2 (*x*, 1/2-*y*, 3/2+*z*) distance of 3.9823 (9) Å; *Cg*2···*Cg*1 (-1/2+*x*, 1/2-*y*, 1-*z*) distance of 3.9823 (9) Å; *Cg*1···*Cg*2 (-*x*, 1-*y*, 1-*z*) distance of 4.2397 (9) Å; *Cg*3···*Cg*1 (1/2-*x*, 1/2+*y*, *z*) distance of 5.0406 (8) Å; where *Cg*1, *Cg*2 and *Cg*3 are the centroids of the rings C1···C6, C10···C15, and N2/C7/N4/C9/N3/C8, respectively.

### Experimental

Melamine (1.261 g, 10 mmol) was dissolved in 200 ml of hot distilled water. 3-Nitrophenol (1.391 g, 10 mmol) was dissolved in 100 ml of distilled water, separately. The 3-nitrophenol solution was added gently to the hot solution of melamine, and the mixture stirred well for nearly five hours to get an homogeneous solution. Water was then allowed to evaporate. Within few days, tiny transparent, yellowish crystals were formed.

### Refinement

H atoms of aromatic CH groups were positioned geometrically and refined using a riding model with C—H = 0.93 Å and *U*_iso_(H) = 1.2*U*_eq_(C). The H atoms bound to O and N atoms were found in a difference map and refined isotropically, with distances restrained to N—H = 0.88 (1) Å and O—H = 0.82 (1) Å (Sheldrick, 2008).

### Crystal data

**Table:**

C_3_H_6_N_6_·2C_6_H_5_NO_3_	*F*(000) = 1680
*M**_r_* = 404.36	*D*_x_ = 1.503 Mg m^−^^3^
Orthorhombic, *P**b**c**a*	Mo *K*α radiation, λ = 0.71073 Å
Hall symbol: -P 2ac 2ab	Cell parameters from 4327 reflections
*a* = 15.5150 (6) Å	θ = 2.3–28.3°
*b* = 12.9137 (6) Å	µ = 0.12 mm^−^^1^
*c* = 17.8323 (6) Å	*T* = 295 K
*V* = 3572.8 (2) Å^3^	Block, yellow
*Z* = 8	0.28 × 0.24 × 0.20 mm

### Data collection

**Table:**

Bruker Kappa APEXII CCD diffractometer	4447 independent reflections
Radiation source: fine-focus sealed tube	3352 reflections with *I* > 2σ(*I*)
Graphite monochromator	*R*_int_ = 0.028
ω and φ scans	θ_max_ = 28.3°, θ_min_ = 2.3°
Absorption correction: multi-scan (*SADABS*; Sheldrick, 1996)	*h* = −20→13
*T*_min_ = 0.967, *T*_max_ = 0.977	*k* = −17→7
19568 measured reflections	*l* = −23→23

### Refinement

**Table:**

Refinement on *F*^2^	Secondary atom site location: difference Fourier map
Least-squares matrix: full	Hydrogen site location: inferred from neighbouring sites
*R*[*F*^2^ > 2σ(*F*^2^)] = 0.042	H atoms treated by a mixture of independent and constrained refinement
*wR*(*F*^2^) = 0.119	*w* = 1/[σ^2^(*F*_o_^2^) + (0.0577*P*)^2^ + 0.7906*P*] where *P* = (*F*_o_^2^ + 2*F*_c_^2^)/3
*S* = 1.03	(Δ/σ)_max_ < 0.001
4447 reflections	Δρ_max_ = 0.24 e Å^−^^3^
295 parameters	Δρ_min_ = −0.20 e Å^−^^3^
8 restraints	Extinction correction: *SHELXL97* (Sheldrick, 2008), Fc^*^=kFc[1+0.001xFc^2^λ^3^/sin(2θ)]^-1/4^
0 constraints	Extinction coefficient: 0.0028 (5)
Primary atom site location: structure-invariant direct methods	

### Fractional atomic coordinates and isotropic or equivalent isotropic displacement parameters (Å^2^)

**Table:**

	*x*	*y*	*z*	*U*_iso_*/*U*_eq_	
C1	0.20295 (11)	0.13223 (11)	0.30457 (8)	0.0472 (4)	
C2	0.18602 (9)	0.11918 (10)	0.22906 (8)	0.0406 (3)	
H2	0.1305	0.1048	0.2123	0.049*	
C3	0.25379 (9)	0.12802 (10)	0.17907 (8)	0.0365 (3)	
C4	0.33676 (9)	0.14713 (12)	0.20566 (9)	0.0475 (4)	
H4	0.3826	0.1521	0.1723	0.057*	
C5	0.35064 (12)	0.15871 (14)	0.28162 (10)	0.0597 (5)	
H5	0.4062	0.1712	0.2989	0.072*	
C6	0.28408 (13)	0.15216 (13)	0.33219 (9)	0.0586 (5)	
H6	0.2935	0.1609	0.3833	0.070*	
C7	0.01234 (8)	0.66741 (10)	0.47227 (7)	0.0337 (3)	
C8	0.08123 (8)	0.82016 (10)	0.49038 (7)	0.0337 (3)	
C9	0.14073 (8)	0.67001 (10)	0.53192 (7)	0.0322 (3)	
C10	−0.15540 (9)	0.60489 (10)	0.68536 (8)	0.0383 (3)	
C11	−0.07322 (10)	0.59736 (15)	0.65597 (9)	0.0546 (4)	
H11	−0.0643	0.5851	0.6052	0.066*	
C12	−0.00506 (10)	0.60861 (16)	0.70459 (10)	0.0595 (5)	
H12	0.0510	0.6040	0.6864	0.071*	
C13	−0.01848 (9)	0.62665 (13)	0.78025 (9)	0.0464 (4)	
H13	0.0283	0.6340	0.8124	0.056*	
C14	−0.10170 (8)	0.63378 (10)	0.80810 (8)	0.0365 (3)	
C15	−0.17126 (8)	0.62232 (10)	0.76020 (8)	0.0369 (3)	
H15	−0.2274	0.6263	0.7782	0.044*	
N1	0.13017 (13)	0.12490 (12)	0.35762 (9)	0.0659 (4)	
N2	0.01376 (7)	0.77043 (9)	0.46051 (6)	0.0361 (3)	
N3	0.14703 (7)	0.77395 (8)	0.52606 (6)	0.0354 (2)	
N4	0.07430 (6)	0.61329 (8)	0.50739 (6)	0.0345 (3)	
N5	−0.05479 (8)	0.61359 (11)	0.44621 (8)	0.0461 (3)	
N6	0.08276 (9)	0.92415 (10)	0.48582 (8)	0.0457 (3)	
N7	0.20470 (8)	0.61983 (10)	0.56654 (8)	0.0445 (3)	
N8	−0.22955 (8)	0.59341 (10)	0.63461 (7)	0.0459 (3)	
O1	0.23651 (6)	0.11610 (8)	0.10455 (6)	0.0433 (3)	
O2	0.14494 (13)	0.13811 (14)	0.42402 (8)	0.1006 (6)	
O3	0.05950 (11)	0.10386 (16)	0.33363 (10)	0.0968 (5)	
O4	−0.11865 (7)	0.65065 (10)	0.88192 (6)	0.0537 (3)	
O5	−0.30194 (7)	0.60094 (10)	0.66114 (7)	0.0583 (3)	
O6	−0.21580 (9)	0.57499 (12)	0.56836 (6)	0.0716 (4)	
H1	0.2744 (10)	0.1481 (14)	0.0807 (10)	0.068 (6)*	
H4A	−0.0754 (9)	0.6682 (15)	0.9062 (9)	0.064 (6)*	
H6A	0.0446 (9)	0.9511 (14)	0.4564 (9)	0.065 (5)*	
H6B	0.1328 (8)	0.9538 (13)	0.4931 (10)	0.061 (5)*	
H7A	0.2051 (10)	0.5516 (7)	0.5665 (9)	0.048 (4)*	
H7B	0.2525 (8)	0.6534 (12)	0.5769 (9)	0.050 (5)*	
H5A	−0.0593 (11)	0.5475 (8)	0.4593 (10)	0.061 (5)*	
H5B	−0.1003 (8)	0.6483 (13)	0.4321 (10)	0.058 (5)*	

### Atomic displacement parameters (Å^2^)

**Table:**

	*U*^11^	*U*^22^	*U*^33^	*U*^12^	*U*^13^	*U*^23^
C1	0.0631 (10)	0.0362 (7)	0.0423 (8)	−0.0015 (7)	0.0035 (7)	0.0051 (6)
C2	0.0372 (7)	0.0385 (7)	0.0461 (7)	−0.0032 (6)	−0.0005 (6)	0.0041 (6)
C3	0.0350 (7)	0.0346 (6)	0.0398 (7)	−0.0048 (5)	−0.0051 (5)	0.0045 (5)
C4	0.0369 (8)	0.0514 (8)	0.0542 (8)	−0.0108 (6)	−0.0080 (7)	0.0107 (7)
C5	0.0587 (10)	0.0596 (10)	0.0608 (10)	−0.0195 (8)	−0.0260 (9)	0.0123 (8)
C6	0.0849 (13)	0.0476 (9)	0.0433 (8)	−0.0136 (8)	−0.0168 (9)	0.0063 (7)
C7	0.0258 (6)	0.0459 (7)	0.0293 (6)	0.0013 (5)	−0.0018 (5)	0.0003 (5)
C8	0.0298 (6)	0.0419 (7)	0.0295 (6)	0.0021 (5)	0.0051 (5)	0.0018 (5)
C9	0.0252 (6)	0.0416 (7)	0.0300 (6)	0.0001 (5)	−0.0010 (5)	0.0011 (5)
C10	0.0370 (7)	0.0373 (6)	0.0405 (7)	−0.0007 (5)	−0.0052 (6)	0.0000 (5)
C11	0.0469 (9)	0.0779 (11)	0.0391 (7)	0.0020 (8)	0.0061 (7)	−0.0013 (7)
C12	0.0330 (8)	0.0935 (13)	0.0520 (9)	0.0002 (8)	0.0107 (7)	−0.0027 (9)
C13	0.0281 (7)	0.0632 (9)	0.0479 (8)	−0.0024 (6)	−0.0015 (6)	−0.0034 (7)
C14	0.0300 (7)	0.0396 (6)	0.0399 (7)	−0.0037 (5)	0.0012 (5)	−0.0049 (5)
C15	0.0271 (6)	0.0389 (6)	0.0447 (7)	−0.0028 (5)	0.0000 (5)	−0.0056 (5)
N1	0.0904 (13)	0.0530 (8)	0.0543 (9)	0.0022 (8)	0.0230 (9)	0.0047 (7)
N2	0.0293 (6)	0.0447 (6)	0.0342 (5)	0.0039 (5)	−0.0022 (4)	0.0046 (4)
N3	0.0287 (5)	0.0406 (6)	0.0370 (6)	−0.0036 (4)	−0.0031 (4)	0.0018 (4)
N4	0.0259 (5)	0.0399 (6)	0.0377 (6)	0.0000 (4)	−0.0052 (4)	0.0012 (4)
N5	0.0306 (6)	0.0515 (7)	0.0561 (7)	−0.0013 (6)	−0.0156 (6)	0.0033 (6)
N6	0.0431 (8)	0.0406 (6)	0.0535 (7)	0.0022 (6)	0.0009 (6)	0.0055 (5)
N7	0.0294 (6)	0.0457 (7)	0.0585 (8)	−0.0017 (5)	−0.0150 (6)	0.0062 (6)
N8	0.0481 (8)	0.0436 (6)	0.0460 (7)	0.0003 (6)	−0.0104 (6)	−0.0007 (5)
O1	0.0350 (5)	0.0563 (6)	0.0386 (5)	−0.0122 (5)	−0.0010 (4)	0.0022 (4)
O2	0.1446 (16)	0.1092 (13)	0.0480 (8)	0.0043 (11)	0.0257 (9)	−0.0016 (8)
O3	0.0729 (10)	0.1297 (15)	0.0876 (11)	−0.0121 (10)	0.0340 (9)	−0.0054 (10)
O4	0.0298 (5)	0.0893 (8)	0.0421 (6)	−0.0117 (5)	0.0014 (4)	−0.0207 (5)
O5	0.0396 (6)	0.0704 (8)	0.0648 (7)	0.0011 (5)	−0.0132 (5)	−0.0101 (6)
O6	0.0719 (9)	0.1012 (10)	0.0417 (6)	0.0024 (8)	−0.0125 (6)	−0.0077 (6)

### Geometric parameters (Å, º)

**Table:**

C1—C6	1.376 (2)	C10—N8	1.4712 (18)
C1—C2	1.382 (2)	C11—C12	1.375 (2)
C1—N1	1.476 (2)	C11—H11	0.9300
C2—C3	1.383 (2)	C12—C13	1.385 (2)
C2—H2	0.9300	C12—H12	0.9300
C3—O1	1.3644 (16)	C13—C14	1.3865 (19)
C3—C4	1.3938 (19)	C13—H13	0.9300
C4—C5	1.380 (2)	C14—O4	1.3599 (16)
C4—H4	0.9300	C14—C15	1.3842 (18)
C5—C6	1.374 (3)	C15—H15	0.9300
C5—H5	0.9300	N1—O3	1.208 (2)
C6—H6	0.9300	N1—O2	1.218 (2)
C7—N5	1.3356 (17)	N5—H5A	0.888 (9)
C7—N4	1.3434 (16)	N5—H5B	0.874 (9)
C7—N2	1.3469 (18)	N6—H6A	0.863 (9)
C8—N2	1.3385 (16)	N6—H6B	0.875 (9)
C8—N3	1.3429 (16)	N7—H7A	0.881 (9)
C8—N6	1.3456 (19)	N7—H7B	0.878 (9)
C9—N7	1.3364 (17)	N8—O5	1.2226 (16)
C9—N4	1.3380 (16)	N8—O6	1.2238 (17)
C9—N3	1.3498 (17)	O1—H1	0.835 (9)
C10—C15	1.3756 (19)	O4—H4A	0.830 (9)
C10—C11	1.382 (2)		
			
C6—C1—C2	123.05 (15)	C11—C12—C13	121.09 (14)
C6—C1—N1	118.84 (15)	C11—C12—H12	119.5
C2—C1—N1	118.10 (15)	C13—C12—H12	119.5
C1—C2—C3	118.25 (14)	C12—C13—C14	120.01 (14)
C1—C2—H2	120.9	C12—C13—H13	120.0
C3—C2—H2	120.9	C14—C13—H13	120.0
O1—C3—C2	117.97 (12)	O4—C14—C15	117.63 (12)
O1—C3—C4	122.19 (13)	O4—C14—C13	122.51 (12)
C2—C3—C4	119.83 (13)	C15—C14—C13	119.86 (13)
C5—C4—C3	119.82 (15)	C10—C15—C14	118.47 (12)
C5—C4—H4	120.1	C10—C15—H15	120.8
C3—C4—H4	120.1	C14—C15—H15	120.8
C6—C5—C4	121.37 (15)	O3—N1—O2	123.13 (18)
C6—C5—H5	119.3	O3—N1—C1	118.81 (16)
C4—C5—H5	119.3	O2—N1—C1	118.04 (19)
C5—C6—C1	117.66 (15)	C8—N2—C7	115.13 (11)
C5—C6—H6	121.2	C8—N3—C9	115.05 (11)
C1—C6—H6	121.2	C9—N4—C7	114.76 (11)
N5—C7—N4	116.71 (12)	C7—N5—H5A	118.0 (12)
N5—C7—N2	118.20 (12)	C7—N5—H5B	117.7 (12)
N4—C7—N2	125.08 (11)	H5A—N5—H5B	120.4 (17)
N2—C8—N3	124.74 (12)	C8—N6—H6A	115.2 (13)
N2—C8—N6	117.95 (12)	C8—N6—H6B	116.3 (12)
N3—C8—N6	117.30 (12)	H6A—N6—H6B	121.5 (19)
N7—C9—N4	117.24 (12)	C9—N7—H7A	119.3 (11)
N7—C9—N3	117.65 (12)	C9—N7—H7B	119.0 (11)
N4—C9—N3	125.09 (11)	H7A—N7—H7B	119.2 (15)
C15—C10—C11	122.99 (13)	O5—N8—O6	123.30 (13)
C15—C10—N8	118.25 (12)	O5—N8—C10	118.19 (12)
C11—C10—N8	118.76 (13)	O6—N8—C10	118.50 (13)
C12—C11—C10	117.58 (14)	C3—O1—H1	107.5 (14)
C12—C11—H11	121.2	C14—O4—H4A	113.1 (13)
C10—C11—H11	121.2		
			
C6—C1—C2—C3	−0.9 (2)	C6—C1—N1—O3	−177.46 (17)
N1—C1—C2—C3	178.75 (12)	C2—C1—N1—O3	2.9 (2)
C1—C2—C3—O1	−179.35 (12)	C6—C1—N1—O2	0.8 (2)
C1—C2—C3—C4	1.5 (2)	C2—C1—N1—O2	−178.81 (16)
O1—C3—C4—C5	179.91 (14)	N3—C8—N2—C7	3.86 (18)
C2—C3—C4—C5	−1.0 (2)	N6—C8—N2—C7	−174.73 (12)
C3—C4—C5—C6	−0.2 (3)	N5—C7—N2—C8	177.70 (12)
C4—C5—C6—C1	0.8 (3)	N4—C7—N2—C8	−3.47 (18)
C2—C1—C6—C5	−0.3 (2)	N2—C8—N3—C9	−1.28 (18)
N1—C1—C6—C5	−179.92 (14)	N6—C8—N3—C9	177.32 (12)
C15—C10—C11—C12	−0.4 (3)	N7—C9—N3—C8	179.61 (12)
N8—C10—C11—C12	179.86 (15)	N4—C9—N3—C8	−2.16 (18)
C10—C11—C12—C13	0.1 (3)	N7—C9—N4—C7	−179.26 (12)
C11—C12—C13—C14	−0.1 (3)	N3—C9—N4—C7	2.50 (18)
C12—C13—C14—O4	179.52 (15)	N5—C7—N4—C9	179.35 (12)
C12—C13—C14—C15	0.3 (2)	N2—C7—N4—C9	0.50 (18)
C11—C10—C15—C14	0.6 (2)	C15—C10—N8—O5	0.72 (19)
N8—C10—C15—C14	−179.63 (12)	C11—C10—N8—O5	−179.50 (14)
O4—C14—C15—C10	−179.81 (12)	C15—C10—N8—O6	−177.97 (14)
C13—C14—C15—C10	−0.6 (2)	C11—C10—N8—O6	1.8 (2)

### Hydrogen-bond geometry (Å, º)

Cg3 is the centroid of the melamine triamine ring.

**Table:**

*D*—H···*A*	*D*—H	H···*A*	*D*···*A*	*D*—H···*A*
O1—H1···N3^i^	0.84 (1)	1.86 (1)	2.6907 (14)	176 (2)
O4—H4*A*···N2^ii^	0.83 (1)	1.87 (1)	2.6876 (14)	170 (2)
N5—H5*A*···N4^iii^	0.89 (1)	2.17 (1)	3.0594 (18)	178 (17)
N5—H5*B*···O1^iv^	0.87 (1)	2.25 (1)	2.9613 (16)	138 (15)
N7—H7*A*···O1^v^	0.88 (1)	2.32 (1)	3.1600 (17)	159 (14)
N7—H7*B*···O4^vi^	0.88 (1)	2.13 (1)	2.9180 (16)	149 (15)
C6—H6···*Cg*3^vii^	0.93	2.95	3.7504 (18)	145

Symmetry codes: (i) −*x*+1/2, −*y*+1, *z*−1/2; (ii) *x*, −*y*+3/2, *z*+1/2; (iii) −*x*, −*y*+1, −*z*+1; (iv) −*x*, *y*+1/2, −*z*+1/2; (v) *x*, −*y*+1/2, *z*+1/2; (vi) *x*+1/2, *y*, −*z*+3/2; (vii) −*x*+1/2, *y*−1/2, *z*.
